# Dr. E. R, Axtell

**Published:** 1900-02

**Authors:** 


Dr. E. R, Axtell, of Denver, Col., died at his home December 14, 1899, of septic infection, following an autopsy, aged 33 years. He was editor of the *Colorado Medical Journal* at the time of his death and besides occupied various hospital positions. He was a greatly respected member of the profession, as is amply attested by resolutions passed by his colleagues in the several societies and other bodies in which he had membership, in some which he had been an officer. The *Colorado Journal* was greatly improved during his editorship. The *Medical News* for January 20, 1900, makes posthumous publication of a paper by Dr. Axtell, entitled Diuretics in renal dropsy; their indications and uses—which is an interesting contribution to the subject.

